# The genetics of rhizosheath size in a multiparent mapping population of wheat

**DOI:** 10.1093/jxb/erv223

**Published:** 2015-05-11

**Authors:** Emmanuel Delhaize, Tina M. Rathjen, Colin R. Cavanagh

**Affiliations:** CSIRO Agriculture, GPO Box 1600, Canberra, ACT 2601, Australia

**Keywords:** Genetics, MAGIC: multiparent advanced generation intercross, rhizosheath, root hairs, roots, wheat.

## Abstract

Rhizosheaths comprise soil that adheres to plant roots and, in some species, are indicative of root hair length. In this study, the genetics of rhizosheath size in wheat was investigated by screening the progeny of multiparent advanced generation intercrosses (MAGIC). Two MAGIC populations were screened for rhizosheath size using a high throughput method. One MAGIC population was developed from intercrosses between four parents (4-way) and the other from intercrosses between eight parents (8-way). Transgressive segregation for rhizosheath size was observed in both the 4-way and 8-way MAGIC populations. A quantitative trait loci (QTL) analysis of the 4-way population identified six major loci located on chromosomes 2B, 4D, 5A, 5B, 6A, and 7A together accounting for 42% of the variation in rhizosheath size. Rhizosheath size was strongly correlated with root hair length and was robust across different soil types in the absence of chemical constraints. Rhizosheath size in the MAGIC populations was a reliable surrogate for root hair length and, therefore, the QTL identified probably control root hair elongation. Members of the basic helix-loop-helix family of transcription factors have previously been identified to regulate root hair length in *Arabidopsis* and rice. Since several wheat members of the basic helix-loop-helix family of genes are located within or near the QTL, these genes are candidates for controlling the long root hair trait. The QTL for rhizosheath size identified in this study provides the opportunity to implement marker-assisted selection to increase root hair length for improved phosphate acquisition in wheat.

## Highlight

Rhizosheath size of wheat was used as a surrogate for root hair length to map QTL in lines derived from multiparent advanced generation intercrosses.

## Introduction

Rhizosheaths comprise of the soil that remains firmly bound to roots (McCully, 1999) and, for some species, are indicative of the length of root hairs. Root hairs are single-celled tubular extensions to plant roots (Datta *et al.*, 2011) and provide structural stability to a rhizosheath which may explain the strong relationship that exists between rhizosheath size and root hair length in some species. For instance, rhizosheath size of young wheat (*Triticum aestivum*) seedlings grown on acid soil was strongly correlated with the length of root hairs (Delhaize *et al.*, 2012). By contrast, George *et al.* (2014) found that rhizosheath size was poorly correlated with root hair length in barley (*Hordeum vulgare*) grown on soil in the absence of constraints and suggested that factors other than root hair length contributed towards the formation of a rhizosheath in this species. However, barley mutants that lack root hairs either lack a rhizosheath or the rhizosheath is substantially smaller than the wild type (Haling *et al.*, 2010; George *et al.*, 2014). Similarly, maize (*Zea mays*) mutants that lack root hairs have considerably smaller rhizosheaths than wild-type plants when grown in soil for up to 60 d (Wen and Schnable, 1994). In addition to a role in the formation of rhizosheaths, root hairs increase the surface area of roots to allow more effective exploration of soil compared with roots that lack these hairs. Although root hairs are not essential for plant growth, studies on mutants that lack root hairs have shown that phosphate uptake from the soil is reduced compared with wild-type plants (Bates and Lynch, 2000; Gahoonia *et al.*, 2002; Gahoonia and Nielsen, 2003; Brown *et al.*, 2012; Haling *et al.*, 2013). Phosphate is poorly mobile in the soil and, as a consequence, its uptake is largely limited by diffusion to the root. A root trait that increases the volume of soil explored will enable more efficient uptake of ions such as phosphate but may not be as effective in improving the efficiency of uptake of more mobile ions such as nitrate.

Rhizosheaths were described over 100 years ago (Volkens, 1987), yet there is a relatively small body of literature on the topic and only recently has the genetics of rhizosheath size been studied. In the first study on the genetics of rhizosheath size, George *et al.* (2014) used a genome-wide association analysis to identify loci associated with large rhizosheaths in barley. By contrast with rhizosheaths, the genetics of root hair formation and development has been extensively studied, particularly in *Arabidopsis thaliana*, with over 140 genes associated with root hair formation and development in this species alone (Kwasniewski *et al.*, 2013). Many of these studies are based on mutants with altered root hair morphology or mutants that lack root hairs altogether. A particularly interesting class of genes identified in several species encodes basic helix-loop-helix (bHLH) transcription factors that control root hair length. Rice mutants of *OsRHL1* have short and deformed root hairs; however, when *OsRHL1* was over-expressed, root hairs elongated up to three times more than those on wild-type roots (Ding *et al.*, 2009). The isolation of a related gene from *Arabidopsis* that also controls the length of root hairs indicates that the mechanism controlling root hair length, based on bHLH transcription factors, may be widespread through the plant kingdom (Yi *et al.*, 2010).

There has been considerably less effort aimed at identifying the genes that control the natural variation in root hair length in crop or pasture species. Studies on maize (*Zea mays*) (Zhu *et al.*, 2005) and common bean (*Phaseolus vulgaris*) (Yan *et al.*, 2004) identified quantitative trait loci (QTL) from biparental mapping populations accounting for a large proportion of the variation in root hair length. Other studies have shown variation in root hair length within species which was correlated with improved phosphorus (P) acquisition efficiency (Gahoonia and Nielsen, 1997, 2004; Krasilnikoff *et al.*, 2003; Wang *et al.*, 2004; Vandamme *et al.*, 2013). Of the few examples where genotypes were successfully bred for increased root hair length, Caradus (1981) showed that white clover lines that differed in root hair length also differed in their ability to take up P from soils that had been sterilized and were, therefore, non-mychorrhizal. The benefit of root hairs on P uptake was abolished on soils that enabled mychorrhizal associations indicating that, for some species, root hairs can be substituted for by the fungus. Similarly, a study of five pasture species showed an inverse relationship between the benefit of mycorrhizal associations on P nutrition and root hair length with the authors concluding that root hairs and external hyphae act as alternate ways of shortening the diffusion path for the uptake of soil phosphate (Schweiger *et al.*, 1995).

Previous studies that screened seedlings for root hair length used methods based on growing seedlings in artificial systems such as paper cigars, hydroponics or on agar with root segments subsequently photographed and root hair length determined by image analysis (Gahoonia and Nielsen, 2004; Zhu *et al.*, 2005; Vandamme *et al.*, 2013). Other studies removed segments of roots from plants grown in soil, either in the field or in pots, and root hair lengths were then measured by image analysis (Wang *et al.*, 2004; Haling *et al.*, 2010). An alternate method, based on screening for rhizosheath size, showed that this trait was strongly correlated with root hair length in wheat grown on acid soil and, therefore, a reliable surrogate for root hair length (Delhaize *et al.*, 2012). Measuring the rhizosheath is a simple assay that can be adapted to high-throughput screens.

Recombinant inbred lines (RILs) developed from multiparent advanced generation intercrosses (MAGIC) provide several advantages over biparental or association mapping populations for QTL analysis. These advantages include the ability to develop high-density genetic maps as a consequence of increased opportunity for recombination and the use of parental germplasm that encompass a large proportion of the variation found in modern cultivars (Cavanagh *et al.*, 2008; Huang *et al.*, 2012). In this study, a rhizosheath screen was used on soil devoid of chemical or physical constraints to assess the variation present in two wheat MAGIC populations, QTL in one of these populations was mapped, and the relationship that exists between rhizosheath size and root hair length was explored.

## Materials and methods

### Germplasm

The development of the 4 way MAGIC population is described by Huang *et al.* (2012). The 8-way MAGIC population was developed by intercrosses between the wheat cultivars Westonia, Yitpi, AC Barrie, Xiaoyan 54, Pastor, Alsen, Baxter, and Volcani. The development and genetic characterization of the 8-way MAGIC population will be described in detail in a future article and a description of the concept using eight parental lines is provided by Cavanagh *et al.* (2008). Sub-populations of the 4-way and 8-way MAGIC RILs were selected for rhizosheath screens based on genome-wide analysis of molecular markers to maximize the allelic diversity of lines used in the rhizosheath assay by utilizing the R package spclust (Huang *et al.*, 2013a). Spica (small rhizosheath) and Maringa (large rhizosheath) were identified as genotypes with contrasting rhizosheath sizes and served as check lines that were included in all rhizosheath screens.

### Rhizosheath screen

Rhizosheaths were screened based on a method described previously by Delhaize *et al.* (2012). Seedlings were planted into 250g of soil in small pots prepared as described below and grown in a controlled growth cabinet set at 23 °C with a 16/8h light/dark regime. To reduce drying of the soil surface, trays of water were placed within the cabinet to maintain the humidity at about 70%. Pre-germinated seed with 3–6mm roots were planted in the soil, moistened, and set up as described below under the heading ‘Soils’. Pots were placed in trays and covered with transparent plastic lids to reduce moisture loss during the experiment. The surface of the pots was moistened on the second day (~10ml) and, after 3 d growth, plants were harvested. Soil was tipped out of the pots and seedlings were gently removed from the soil. Seedlings were assayed only if their shoots had reached a minimum length of 3cm. The three primary seminal roots were cut off directly into a small tray, weighed with the adhering soil still intact, and then their lengths were measured. Rhizosheaths are expressed as g m^–1^ of root, and included the weight of both the fresh root and the moist soil. The fresh weight of the roots comprised only 5.0±0.2% (mean of 20 seedlings) of the total weight of the rhizosheath indicating that variations in root fresh weight would be a minor contributor to any variation in rhizosheath size. Furthermore, root diameter as an indicator of root fresh weight, was relatively constant across 4-way parental lines and a random selection of MAGIC 4-way RILs (see Supplementary Fig. S1A at *JXB* online) indicating that fresh root weight was unlikely to vary significantly across the various lines. Furthermore, since fresh weight contributed 5% of the weight of the rhizosheath, this indicates that genetic variation for root weight was unlikely to interfere with the QTL analysis and that expressing the rhizosheath on a per length basis was an appropriate measure.

The screening was conducted in ‘Sets’ (two Sets for 4-way RILs; one Set for 8-way RILs) where ~230 entries (MAGIC lines plus checks comprising of the cultivars Spica and Maringa) were screened in each Set. Each Set consisted of six replicates. Within each replicate there were two batches of six trays, with each tray comprising pots in a rectangular arrangement of five rows by four columns. The ASreml 3 package (VSN International) was used to analyse rhizosheath data. The linear mixed model associated with the non-genetic effects reflected the experimental designs for each Set, taking particular care to include terms which accommodate the experimental and observational units. The effects for check lines and parental lines, as well as the Set effects, were fitted as fixed effects in order to exclude them from sources of variation associated with the genetic effects. The remainder of the experimental design terms were included as random effects for each Set.

Selected lines from the tails of the rhizosheath size distributions of both the 4-way and 8-way screens were re-analysed along with parental lines using six replicates in randomized block designs within a cabinet. After rhizosheath size was measured, a 5mm segment of root was excised from the primary root at a distance of one-quarter of the length down the root below the seed and root hair length was assayed as described previously (Delhaize *et al.*, 2012). For experiments that assessed rhizosheath size in different soil types, the same lines were assayed across the different soils using six replicates for each soil and experiments were arranged in random block designs.

### Rhizoboxes

Rhizoboxes were used as an alternate method for measuring root hair length. The method allowed root hairs to be measured on plants grown for up to 11 d, during which lateral roots were well developed. The rhizoboxes were built from Perspex^TM^ and had internal dimensions of 26×20×1cm with the front comprising a clear face whereas other faces were opaque. Two types were constructed—one with a horizontal (landscape) orientation and one with a vertical (portrait) orientation. Seedlings grown for up to 6 d were sown into the landscape rhizoboxes and those grown for 11 d were sown into the portrait rhizoboxes. The rhizoboxes were filled with Robertson soil (see below) moistened to 90% field capacity, and then packed to the same bulk density (~0.8g cm^–3^) as used in the rhizosheath screens, sown with pre-germinated seeds (two for landscape and one for portrait boxes), and maintained at an angle of about 45° during the growth of the seedlings so that roots grew along the clear front face of the rhizobox. At various times, sections of roots were photographed through the clear face of the rhizobox at ×2.5 magnification with the resulting image being analysed for root hair length as described previously by Delhaize *et al.* (2012).

### Soils

For most rhizosheath assays, a soil from the Robertson region of New South Wales, Australia was used due to its ease of manipulation and water-holding capacity. The soil is a Ferrosol and, in its native state, is acidic (pH 4.4) and Al^3+^ toxic. Prior to use, the soil was neutralized with 30g kg^–1^ of CaCO_3_ to yield a final pH of 6.2 in a 0.01M CaCl_2_ extract. Soil moisture was measured before each experiment and adjusted to 90% field capacity. To assess the influence of soil moisture on rhizosheath size, seedlings were grown in preliminary experiments where soil moisture was varied from 80% to 100% field capacity. Soil moisture had a large effect on the rhizosheath size of maize (Watt *et al.*, 1994) but, for wheat grown over a tighter range of moisture contents of 85–100%, it was found that rhizosheath size remained constant (see Supplementary Fig. S1B at *JXB* online). Seedlings grown on soil with an 80% moisture content had shorter roots (data not shown) and the rhizosheath sizes of the two cultivars assayed did not differ from one another (see Supplementary Fig. S1B at *JXB* online). To reduce further any changes in soil moisture that could have occurred over the 3 d of seedling growth, the surface of the pots was moistened on the second day with 10ml of water, trays of water were included to maintain the humidity at about 70% and the seedling trays were covered with plastic covers. Using these procedures soil moisture content was maintained within the range of 85–95% field capacity over the course of the experiments, well within the range where soil moisture did not affect rhizosheath size when expressed on a per root length basis. Other soils used to assess the robustness of the ranking of rhizosheath size across different soil types included a Calcarosol obtained from Port Kenny in South Australia (pH 7.8; pasture soil) and a Yellow Chromosol from Young, New South Wales, Australia (pH 5.1; cropping soil). Additional properties of these soils are described in Ryan *et al.* (2014) and the soils were classified according to the Australian Soil Classification System (Isbell, 1996). No nutrients were added to the Ferrosol and Yellow Chromosol whereas the Calcarosol was fertilized as described by Ryan *et al.* (2014) and included a medium rate of P (10mg P kg^–1^). Soils were moistened so as to provide adequate water for growth while remaining sufficiently friable so that they could easily be sieved. In this regard, the soils differed markedly and were moistened to 90% field capacity for the Ferrosol, 67% field capacity for the Calcarosol, and 51% field capacity for the Yellow Chromosol. All soils were mixed well after the addition of water, sieved through a 4mm mesh prior to use, and then packed into pots to a bulk density of approximately 0.8g cm^–3^.

### Genetic analyses

Wheat DNA was analysed on the 90K single nucleotide polymorphism (SNP) chip as described previously using Infinium iSelect SNP assays (Cavanagh *et al.*, 2013; Wang *et al.*, 2014) Co-located markers (at the same position on the map) were removed prior to detecting QTL using MPWGAIM (Verbyla *et al.*, 2014).

To identify *bHLH* genes as candidates underlying individual QTL, the rice *OsRHL1* gene was used in a BLASTN search of survey sequences available at the International Wheat Genome Sequencing Consortium (IWGSC) website (http://wheat-urgi.versailles.inra.fr). A chromosome-based draft sequence of the hexaploid wheat genome was recently published in which the construction of genome zippers for chromosomal arms is described (IWGSC, 2014). Genome zippers (version 5) for chromosome arms that possessed QTL for rhizosheath size were downloaded from the IWGSC website and markers flanking the QTL were located on the zipper, although not all flanking markers were present within the zippers. The locations of the *OsRHL1* homologues were identified by searching for wheat contigs within the zippers that contained the relevant sequences.

## Results

**Fig. 1.**
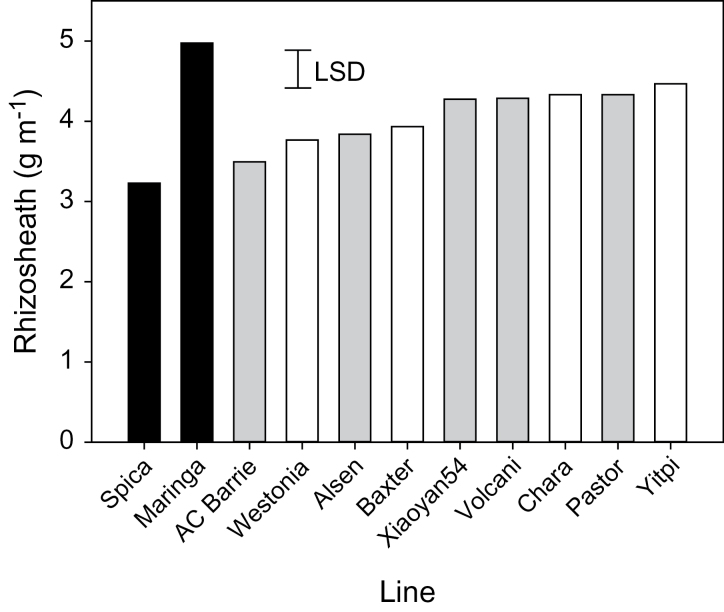
Rhizosheath size of the parental lines of the 4-way (white bars) and 8-way MAGIC populations (grey and white bars excluding the line Chara) and of the check lines Spica and Maringa (black bars). The data show the mean of six replicates with the least significant difference (LSD) for *P* <0.05. Seedlings were grown in a randomized block design in a cabinet for 3 d in a Ferrosol soil limed to pH 6.2.

**Fig. 2.**
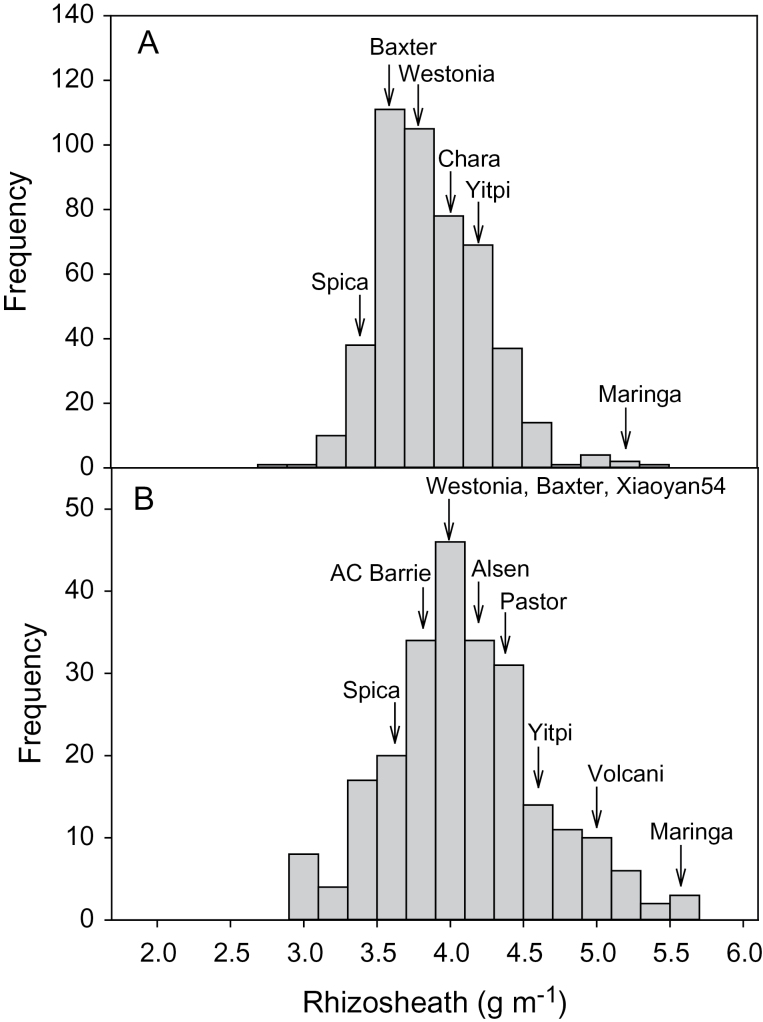
Frequency distributions of rhizosheath size for 466 lines of the 4-way (A) and 230 lines of the 8-way (B) MAGIC populations. The rhizosheath categories of parental and check lines are indicated by the arrows. Data are presented as the number of lines in each rhizosheath size category incrementing by 0.2g m^–1^ (the values for each line is the mean of *n*=4–6 seedlings).

The parental lines of both the 4-way and 8-way MAGIC RILs showed relatively little variation for rhizosheath size and were intermediate between the check lines Spica and Maringa (Fig. 1). Analysis of both the 4-way and 8-way RILs showed evidence of transgressive segregation for rhizosheath size with the largest rhizosheaths similar to those of Maringa and numerous lines with rhizosheaths smaller than Spica (Fig. 2). The 4-way RILs varied by 1.9-fold between the largest and smallest rhizosheaths whereas the variation between parental lines was only 1.2-fold. Similarly, the 8-way RILs varied by 2.2-fold whereas parental lines varied by only 1.3-fold. The two populations screened included Westonia, Yitpi, and Baxter as parents in common as well as the same check lines Spica and Maringa. The average of the 4-way RILs was 3.79±0.35g m^–1^ compared with 4.01±0.52g m^–1^ (mean ±standard deviation) for the 8-way RILs. The common parental and check lines were also, on average, larger for the 8-way screen but their relative rankings for rhizosheath size were similar for both screens (Fig. 2).

Since both populations showed a similar fold variation between the largest and smallest rhizosheath lines, we focused on the 4-way population for QTL analysis because the 8-way RILs would have required much larger numbers to be screened to obtain reliable QTL. Heritability of rhizosheath size was high for both populations with an estimate of 0.74 for the 4-way population and 0.80 for the 8-way population. Six QTL with logarithm of the odds (LOD) values ranging from 4.79 to 9.65 were identified in the 4-way population and, combined, these accounted for about 42% of the variation in rhizosheath size (Table 1). Other minor QTL (LOD <3.0) were also identified (see Supplementary Table S1 at *JXB* online) and together with the major QTL, accounted for 66% of the variation in rhizosheath size. From an estimate of founder effects, it was apparent that parental lines contributed both positive and negative alleles towards rhizosheath size. For example, despite Baxter generally ranking the lowest of the parental lines, it had the largest contribution to the locus on chromosome 7A which accounted for about 9.7% of the variation (Table 1). Conversely, Baxter contributed the largest negative value for the locus on chromosome 5AL. The other parental lines similarly contributed large positive alleles at some loci and large negative alleles at other loci.

**Table 1.** Locations of QTL (LOD >3.0) for rhizosheath size in the 4-way RILs and corresponding founder effects

Chromosomal location	Molecular marker	Distance (cM)	LOD	% var.^*a*^	Founder effects (g m^–1^)			
					Baxter	Chara	Westonia	Yitpi
2BL	Tdurum_contig14482_423	101.4	4.80	5.3	0.040	–0.022	0.113	–0.132
4DS	wsnp_Ex_rep_c67296_65839761	31.8	4.79	4.5	–0.040	–0.115	0.119	0.035
5AL^*b*^	IACX5879	127.3	4.90	5.6	–0.124	0.071	–0.059	0.11
5BL^*b*^	BS00068710_51	138.6	5.92	7.2	–0.007	0.177	–0.132	–0.04
6AL^*b*^	IAAV7384	75.4	9.65	9.4	0.024	-0.123	–0.079	0.176
7AL	BobWhite_rep_c49790_351	124.2	9.62	9.7	0.175	–0.075	0.051	–0.153

^*a*^ The percentage of the variation contributed by each QTL.

^*b*^ Chromosome arms where homologues of *OsRHL1*, a rice *bHLH* gene, are located.

**Fig. 3.**
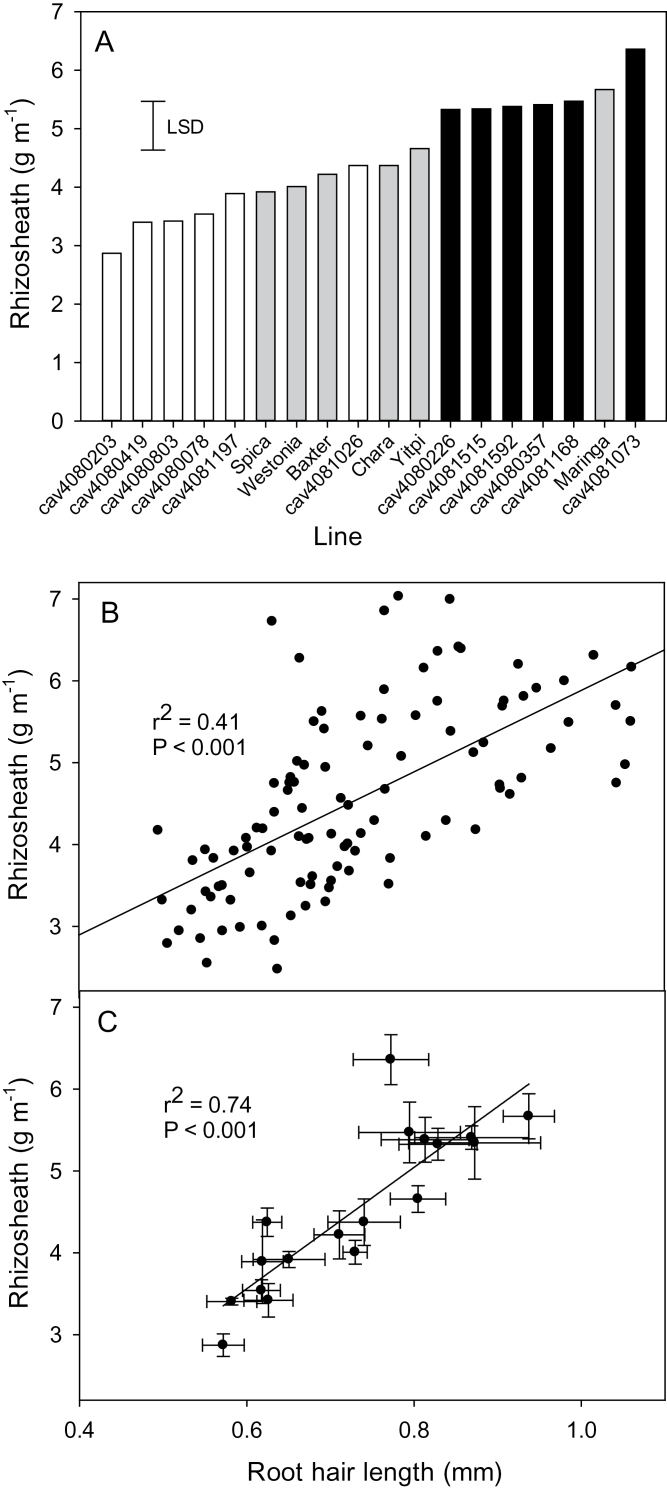
(A) Rhizosheath size is correlated with root hair length in the MAGIC 4-way lines. Rescreen of the MAGIC 4-way lines with six large (black bars) and six small rhizosheath lines (white bars) identified from Fig. 2A. All lines were screened together with six replicates for each line and included parental and reference lines (grey bars). The least significant difference (LSD) for *P*=0.05 is shown. (B). Relationship between root hair length and rhizosheath size plotting values for each individual seedling. (C) Relationship between root hair length and rhizosheath size plotting mean values for each line (*n*=6 for each line, error bars indicate standard errors in both directions).

**Fig. 4.**
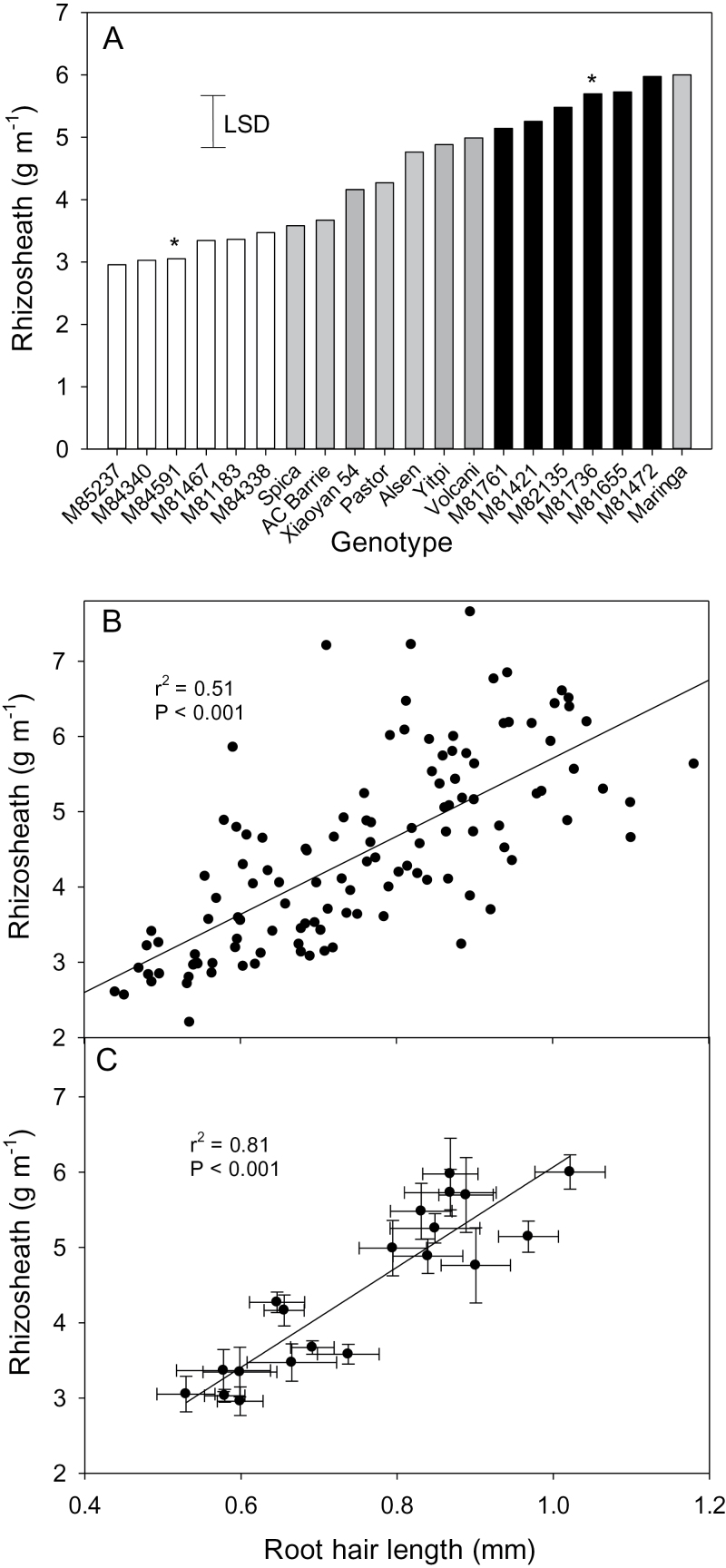
(A) Rhizosheath size is correlated with root hair length in the MAGIC 8-way lines. Rescreen of the MAGIC 8-way lines with the six large (black bars) and the six small rhizosheath (white bars) lines identified from Fig. 2B. All lines were screened together with six replicates and included parental and reference lines (grey bars). The least significant difference (LSD) for *P*=0.05 is shown. (B) Relationship between root hair length and rhizosheath size plotting values for each individual seedling. (C) Relationship between root hair length and rhizosheath size plotting mean values for each line (*n*=6 for each line, error bars indicate standard errors in both directions). The asterisks denote the lines grown in the rhizoboxes shown in Fig. 6 and data of Table 2.

To assess the reproducibility of the rhizosheath assay, lines selected within the tails of the distribution curves were re-assayed. For both populations, the re-assay showed that the small rhizosheath selections were well separated from the large rhizosheath selections with parental lines generally intermediate between these two groups (Figs 3A, 4A). The relationship between root hair length and rhizosheath size was determined by taking root segments for root hair measurements from the same plants that had been measured for rhizosheath size. Plotting the data derived from individual plants showed a significant relationship for both populations with coefficients of determination (*r* ^2^) of 0.41 and 0.51 (Figs 3B, 4B). This relationship was further strengthened when the mean values for rhizosheath and root hair length for each line were plotted with *r* ^2^ increasing to 0.74 and 0.81 (Figs 3C, 4C).

**Fig. 5.**
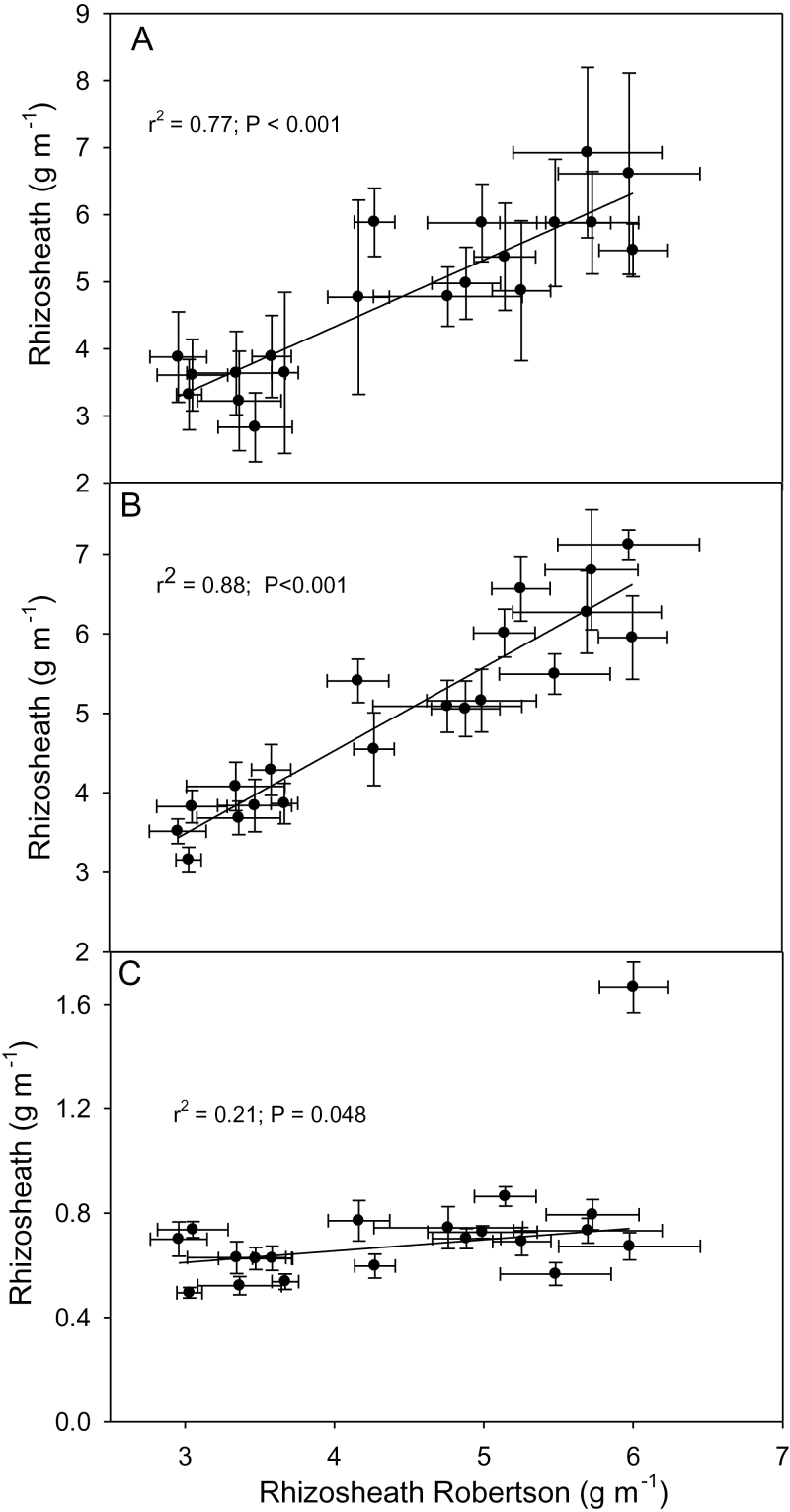
Relationships between rhizosheath size measured on Robertson soil with those measured on three other soils. (A) Robertson (pH 6.2) versus Young (mildly acid, pH 5.1). (B) Robertson versus Port Kenny (alkaline, pH 7.8). (C) Robertson soil (pH 6.2) versus unamended Robertson soil (acidic, pH 4.4 and Al^3+^ toxic). In all cases, six replicates of the lines from Fig. 4 were used in the screens with the means plotted on the graphs. For (C), the outlier value (Maringa) was omitted from the linear regression analysis.

To assess the robustness of the rhizosheath phenotype in different soil types, a selection of lines in three other soils with contrasting properties were assayed (Fig. 5). Rhizosheath sizes of seedlings grown in both the Port Kenny and Young soils were strongly correlated with the rhizosheath size of seedlings grown in the Robertson soil despite one soil being mildly acidic (Young, pH 5.1) and the other alkaline (Port Kenny, pH 7.8). By contrast, rhizosheath sizes of all seedlings were severely reduced when grown in an acid, Al^3+^-toxic soil (unamended Robertson, pH 4.4) and there was a poor relationship with the rhizosheath size of seedlings grown in the Robertson soil adjusted to pH 6.2. Maringa was notable in having the largest rhizosheath when grown on the Al^3+^-toxic soil (Fig. 5C) and this line of Brazilian origin was previously identified as being able to maintain a rhizosheath when grown on acid soil (Delhaize *et al.*, 2012).

**Fig. 6.**
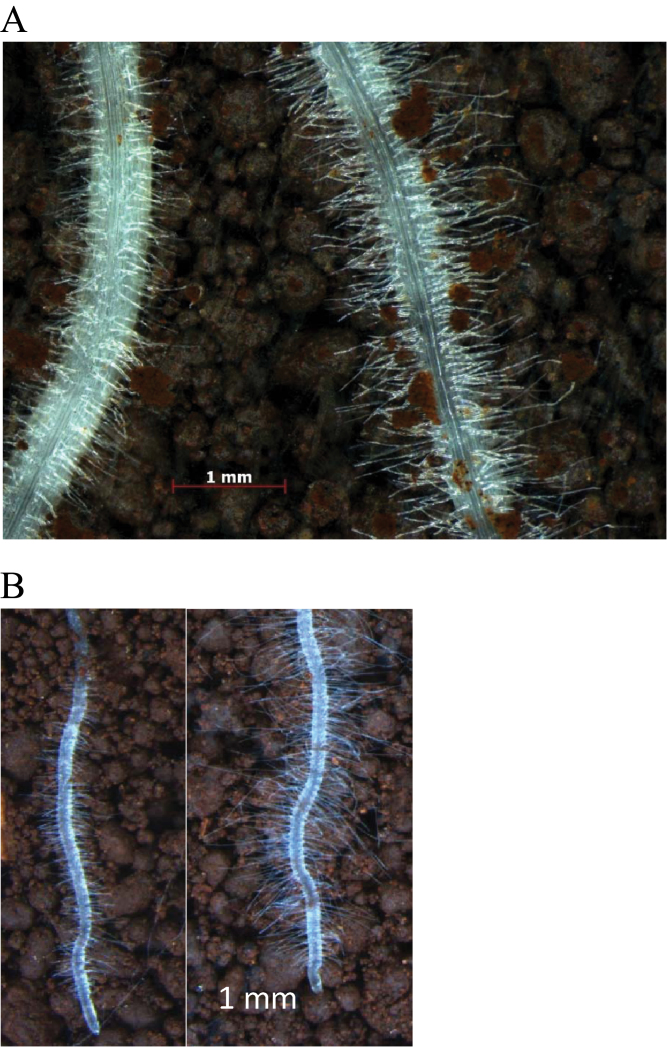
Root hair phenotypes of two RILs with contrasting rhizosheath sizes selected from the 8-way MAGIC screen. In (A) primary seminal roots of seedlings grown for 3 d were photographed approximately half way down the root and (B) is a composite image of the same lines showing lateral roots after 11 d growth. In both images, the root on the left is from line M84591 and the one on the right is from line M81736. Seedlings were grown in limed Robertson soil within a Perspex^TM^ box that was oriented at 45° from the vertical so that the roots were pushed up against the clear surface of the box.

As an alternate method of measuring root hair length, rhizoboxes filled with soil were used whereby roots grew along the clear face of one of the surfaces of the box. This allowed regions of the root to be photographed and the length of hairs measured on roots of seedlings grown for up to 11 d. Typically root hairs protruded directly away from the root and were easier to measure than on roots grown embedded in the soil where the hairs became convoluted as they grew around soil particles (Fig. 6). A pair of lines was used to develop the method and to relate root hair length to rhizosheath size. The lines were derived from the tails of the distribution of the 8-way MAGIC lines and differed in rhizosheath size as well as root hair length when a segment of root grown in potted soil was assayed (Fig. 4). Root hairs measured on roots grown for 3 d in the rhizobox were clearly different between genotypes and were of similar lengths to those measured when roots were embedded within soil (Fig. 6; Table 2). Differences in root hair length between genotypes were maintained over 11 d growth and were also apparent on lateral and secondary seminal roots (Fig. 6; Table 2).

**Table 2.** Analysis of root hair length of two wheat lines with contrasting rhizosheaths grown in rhizoboxes and pots

	**Lines**	
	M81736	M84591
	**Rhizosheath size** (g m^–1^)^*a*^	
	4.52 (0.18)	2.91 (0.13)
**Root type and growth period**	**Root hair length** (mm)^*a*^	
Primary seminal 3 d rhizobox	0.98 (0.08)	0.49 (0.03)
Primary seminal 3 d pots	0.89 (0.03)	0.53 (0.04)
Secondary seminal 3 d rhizobox	1.19 (0.10)	0.47 (0.02)
Primary seminal 6 d rhizobox	0.99 (0.07)	0.43 (0.02)
Secondary seminal 6 d rhizobox	1.20 (0.07)	0.43 (0.02)
Primary seminal 11 d rhizobox	1.44 (0.01)	0.52 (0.05)
Lateral 11 d rhizobox	1.04 (0.11)	0.32 (0.05)

^*a*^ Mean and standard error in parenthesis (*n*=12 for rhizosheath assay; *n*=10–11 for roots grown in rhizoboxes for 3 or 6 d; *n*=6 for roots grown for 3 d in pots; *n*=3 for roots grown in rhizoboxes for 11 d).

## Discussion

In this study, it was found that, despite parental lines of the 4-way MAGIC population having relatively small variation in rhizosheath size, the progeny showed transgressive segregation for the trait and QTL could be mapped with confidence. Six major QTL accounted for about 42% of the variation in rhizosheath size with two of the QTL each contributing over 9% of the variation. One QTL was located near the *Rht-D1* locus on chromosome 4D suggesting that this major dwarfing gene affected rhizosheath size. However, no equivalent QTL was identified on chromosome 4B where another major dwarfing gene (*Rht-B1*) is located and these MAGIC RILs are known to be segregating for *Rht-B1* (Huang *et al.*, 2012). The *Rht* genes have previously been shown to have major effects on plant form such as influencing coleoptile length, plant height (Rebetzke *et al.*, 2014), and root length but did not appear to affect root architecture (Wojciechowski *et al.*, 2009). Our data from the 4-way MAGIC RILs suggest that root hair length is also not affected by the dwarfing genes but that one of the QTL was located at a closely linked locus to *Rht-D1*. The parental cultivars Westonia and Yitpi both possess the *Rht-D1* mutation conferring a semi-dwarf habit yet donated a locus for a large rhizosheath in the *Rht-D1* genomic region (Table 1). It is concluded that if the *Rht-D* locus was influencing rhizosheath size then the wild-type *Rht-D* allele would have contributed towards a smaller rhizosheath.

There was a strong relationship between rhizosheath size and length of root hairs indicating that length of root hairs was a major determinant of rhizosheath size in both MAGIC populations. A similar strong relationship was found for root hair length of wheat grown in acid soil where differences in length could be attributed to differences in Al^3+^ tolerance of root hairs between genotypes (Delhaize *et al.*, 2012). It is unlikely that the same QTL are responsible for both Al^3+^ tolerance of root hairs and length of root hairs on plants grown in soil free of constraints. The poor relationship between rhizosheath size of seedlings grown on an Al^3+^-toxic soil with rhizosheath size of seedlings grown in the same soil that had been amended with lime to pH 6.2 (Fig. 5C) supports this notion. This screen and one described previously (Delhaize *et al.*, 2012) included the parental lines of the 4-way and 8-way MAGIC populations and all had small rhizosheaths suggesting that there was little genetic variation for rhizosheath size in acid soil within the parental lines. Another study found that rhizosheath size was positively correlated with the root cylinder volume, calculated based on the annulus formed by the length of hairs surrounding wheat and barley roots (Haling *et al.*, 2010). In those experiments, root hair length was affected by Al^3+^ toxicity and the relationship was established from a series of liming treatments resulting in soils that varied in level of Al^3+^ toxicity.

The strong relationships found between rhizosheath size and root hair length in wheat when grown in both acid and near-neutral soils indicates that rhizosheath size can be used as a surrogate for root hair length in the germplasm analysed. Measuring root hair length directly is slow and cumbersome whereas rhizosheaths can easily be measured and experiments arranged as undertaken here for a relatively high throughput. However, it is likely that factors other than root hair length also contribute to rhizosheath size in wheat. In the relationship shown in Fig. 3C one of the MAGIC RILs is an outlier with a larger rhizosheath than would have been expected based on root hair length alone. It is possible that some other factor, such as greater mucilage production, contributes to the size of the rhizosheath in this line. Nevertheless, measuring rhizosheath size is a useful method to screen for root hair length allowing for a greater throughput than methods that directly measure root hair length. Any seedlings identified by an initial rhizosheath screen can be subsequently assayed for root hair length to verify the phenotype. The rhizosheath trait was robust across soil types that had no known constraints to root growth (e.g. Al^3+^ toxicity) with strong relationships found between rhizosheath size of seedlings grown on the pH 6.2 Robertson soil with rhizosheath size of seedlings grown on alkaline (Port Kenny) or mildly acid (Young) soils (Fig. 5). By contrast, when screened on a Robertson acid soil with known Al^3+^ toxicity, all genotypes had markedly reduced rhizosheaths and the relationship with rhizosheath size screened on the pH 6.2 Robertson soil was poor. A pair of lines differing in rhizosheath size were grown in a rhizobox and the differences in root hair length identified after 3 d growth were maintained over 11 d and were apparent on both lateral and seminal roots. This indicates that, at least for these lines, the phenotype was stable and occurred throughout the root system of young seedlings.

The only other study that has explored the genetics of rhizosheath size is that of George *et al.* (2014) where they identified loci for specific rhizosheath size (g of soil g^–1^ of root) on chromosomes 5H (LOD 3.65) and 7H (LOD 3.16) by genome-wide association mapping of a diverse population of barley genotypes that spanned about a 5-fold variation in rhizosheath size. A locus identified on chromosome 2H (LOD 4.47) was associated with absolute rhizosheath weight (total soil adhering to the root system) but, since it was not associated with specific rhizosheath weight, it suggests that the locus is associated with a large root system. Our data are expressed as amount of soil adhering per unit length of root and is a similar measure to the specific rhizosheath size reported by George *et al.* (2014). Rhizosheath size was poorly correlated with root hair length in barley and, in this case, rhizosheath size would not be a useful surrogate if the aim is to screen for root hair length. Root hairs of the barley lines screened were considerably longer than those of wheat with the majority of lines having hairs longer than 1mm and the longest were up to 2.5mm. It is possible that the rhizosheath surrounding roots that possess hairs longer than 1mm was unstable and prone to disintegrating during measurements resulting in a poor relationship. Alternatively, another factor such as mucilage production could be the main driver of rhizosheath size in barley.

Although rhizosheaths are thought to be important for nutrient uptake and maintaining a moist environment around roots, there is no direct evidence to date that they are beneficial to agricultural plants beyond the benefits contributed by root hair length. For instance, mutant barley lines with small rhizosheaths were compromised for P-accumulation and growth on P-deficient soils but these lines also had short root hairs (George *et al.*, 2014). The effect that root hair length has on P nutrition is established not only from studies that used root hair mutants but also from studies using natural variation within a species that correlated root hair length with P uptake both in pot and field trials (Gahoonia and Nielsen, 1997, 2003, 2004; Bates and Lynch, 2000; Gahoonia *et al.*, 2002; Krasilnikoff *et al.*, 2003; Wang *et al.*, 2004; Vandamme *et al.*, 2013). Mutant barley lines with moderately-sized rhizosheaths did not differ in growth or P accumulation compared to those with large rhizosheaths (approximately double the size) suggesting that rhizosheath size beyond a certain value does not affect P nutrition of barley regardless of the basis for the difference in rhizosheath size (George *et al.*, 2014). In addition, soil properties such as bulk density and particle size can affect root hair length and probably influence the ability of roots to acquire P on soils with different characteristics (Haling *et al.*, 2014). Whether the properties of rhizosheaths contributed by different quantities or the chemical composition of mucilage affect the uptake of mineral nutrients or water relations remains to be established.

On the basis of the strong relationship between root hair length and rhizosheath size in the wheat germplasm screened in this paper, it can be inferred that the QTL are largely associated with controlling the length of root hairs. To date, the genes that contribute to the natural variation in root hair length of cereals are not known. However, mutants lacking root hairs or mutants with short root hairs have been described in cereals with only a few of the mutated genes identified (Hochholdinger *et al.*, 2008; Huang *et al.*, 2013b; Kwasniewski *et al.*, 2013). Of the genes identified, a candidate that may be responsible for controlling a proportion of the natural variation in root hair length is *OsRHL1* which encodes a bHLH transcription factor (Ding *et al.*, 2009). When over-expressed in rice, root hairs were increased by up to 3-fold in transgenic lines compared with wild-type plants. It is plausible that natural variation in expression of wheat orthologues of *OsRHL1* underlie one or more of the identified QTL. This class of genes appears to be widespread in plant species with homologues involved in root hair formation identified in *Arabidopsis* (Menand *et al.*, 2007; Yi *et al.*, 2010) and lotus (Karas *et al.*, 2009). In particular, the *RSL4* gene when over-expressed in *Arabidopsis* increased the final length of root hairs by up to 2.5-fold as a consequence of an extended period of growth rather than an increased rate of growth and is thought to be a master regulator of root hair length in this species (Yi *et al.*, 2010).

The choice was made to focus on identifying wheat homologues to *OsRHL1* since this gene has been shown to be involved in root hair elongation in a cereal species. When *OsRHL1* was used in a BLASTN search of the survey sequences of the IWGSC, some of the strongest hits were located on chromosome arms where major QTL for rhizosheath size were located (Table 1; see Supplementary Table S2 at *JXB* online). Markers in the region of the QTL on chromosomes 5AL, 5BL, and 6AL were identified on the respective genome zippers from the IWGSC and compared with the positions of the homologues identified from the BLAST search. For chromosomes 5BL and 6AL, contigs containing *OsRHL1* homologues were located near the markers suggesting that these *bHLH* genes could underlie the QTL (see Supplementary Data S2 and S3 at *JXB* online). In the case of chromosome 5AL, the contig containing the putative *bHLH* homologue was not present in the genome zipper but one of the QTL markers was a partial sequence of a *bHLH* gene with weak homology to *OsRHL1* and is conceivably a candidate that underlies the locus (see Supplementary Data S1 at *JXB* online). However, the *bHLH* genes are a large family in plants (Feller *et al.*, 2011) and the proximity of *bHLH* genes more distantly related to *OsRHL1* to the 5AL locus may simply be coincidental. Further research will be required to establish whether the *bHLH* homologues are responsible for any of the QTL identified in our study.

It is shown here that rhizosheath size varied within the progeny generated by inter-crosses between four current wheat cultivars used in Australia. Marginally greater variation in rhizosheath size was found in the progeny of the 8-way intercross despite the more diverse genetic backgrounds of the parental lines. As noted above, root hair length is positively correlated with P uptake and is an attractive trait for improving the P acquisition efficiency of wheat. Molecular markers for the QTL conferring positive alleles for rhizosheath size identified in this study could be used directly in breeding programmes. The use of existing cultivars facilitates the breeding process and avoids the risk of introducing more divergent germplasm that may possess genes linked to the rhizosheath trait with detrimental effects on yield or grain quality. However, any benefits of longer root hairs need to be established based on comparing lines, ideally near isogenic lines, that vary in root hair length in pot or field trials with a range of P supplies. It is possible that the benefits of root hairs are only apparent up to a certain length after which no further benefit is conferred to the plant or that properties such as high soil strength restrict the benefits of root hairs. The availability of molecular markers is a useful tool for developing near isogenic lines of wheat that vary in length of root hairs, particularly if alleles at multiple loci need to be combined. The identification of heterogeneous inbred families derived from individual lines in the MAGIC population that are heterozygous for alleles of the major QTL can be used rapidly to generate near isogenic lines differing at a single loci as discussed by Huang *et al.* (2012). Alternatively, if the existing variation for root hair length is insufficient in current cultivars or if alleles with greater effect are needed, the rhizosheath screening method and molecular markers identified in this paper provide valuable tools for assessing more diverse germplasm.

## Supplementary data

Supplementary data can be found at *JXB* online.

Supplementary Fig. S1. Root diameters of the parental lines and a set of random RILs taken from the 4-way MAGIC population (A) and influence of soil moisture content on rhizosheath size (B).

Supplementary Table S1. Sequences producing significant alignments to *OsRHL1* with a BLASTN search of the IWGSC chromosomal arm survey sequences.

Supplementary Table S2. All QTL identified as contributing to formation of rhizosheaths including those with LOD <3.0.

Supplementary Data S1. Genome zipper (v5) from the IWGSC for chromosome arm 5AL showing the locations of markers linked to a QTL for rhizosheath size and the locations of wheat contigs containing wheat homologues of *OsRHL1*.

Supplementary Data S2. Genome zipper (v5) from the IWGSC for chromosome arm 5BL showing the locations of markers linked to a QTL for rhizosheath size and the locations of wheat contigs containing wheat homologues of *OsRHL1*.

Supplementary Data S3. Genome zipper (v5) from the IWGSC for chromosome arm 6AL showing the locations of markers linked to a QTL for rhizosheath size and the locations of wheat contigs containing wheat homologues of *OsRHL1*.

Supplementary Data
