# Supplementary material for: The genetics of rhizosheath size in a multiparent mapping population of wheat
Source: J Exp Bot. 2015 May 11;66(15):4527–36. doi: 10.1093/jxb/erv223 (PMC4507764; doi:10.1093/jxb/erv223)
Supplement: Supplementary Data [file supp_66_15_4527__index.html]

The genetics of rhizosheath size in a multiparent mapping population of wheat — The genetics of rhizosheath size in a multiparent mapping population of wheat — Supplementary Data 

# The genetics of rhizosheath size in a multiparent mapping population of wheat

## Supplementary Data

Data files

**Files in this Data Supplement:**

- Supplementary Data - Supplementary Data
- Supplementary Data - Supplementary Data
