# Supplementary material for: The genetics of rhizosheath size in a multiparent mapping population of wheat
Source: J Exp Bot. 2015 May 11;66(15):4527–36. doi: 10.1093/jxb/erv223 (PMC4507764; doi:10.1093/jxb/erv223)
Supplement: Supplementary Data [file supp_erv223_jexbot144915_file001.pdf]

The genetics of rhizosheath size in a multiparent mapping population of wheat

Emmanuel Delhaize, Tina M. Rathjen and Colin R. Cavanagh

Supplementary Figure S1 and Tables S1 and S2

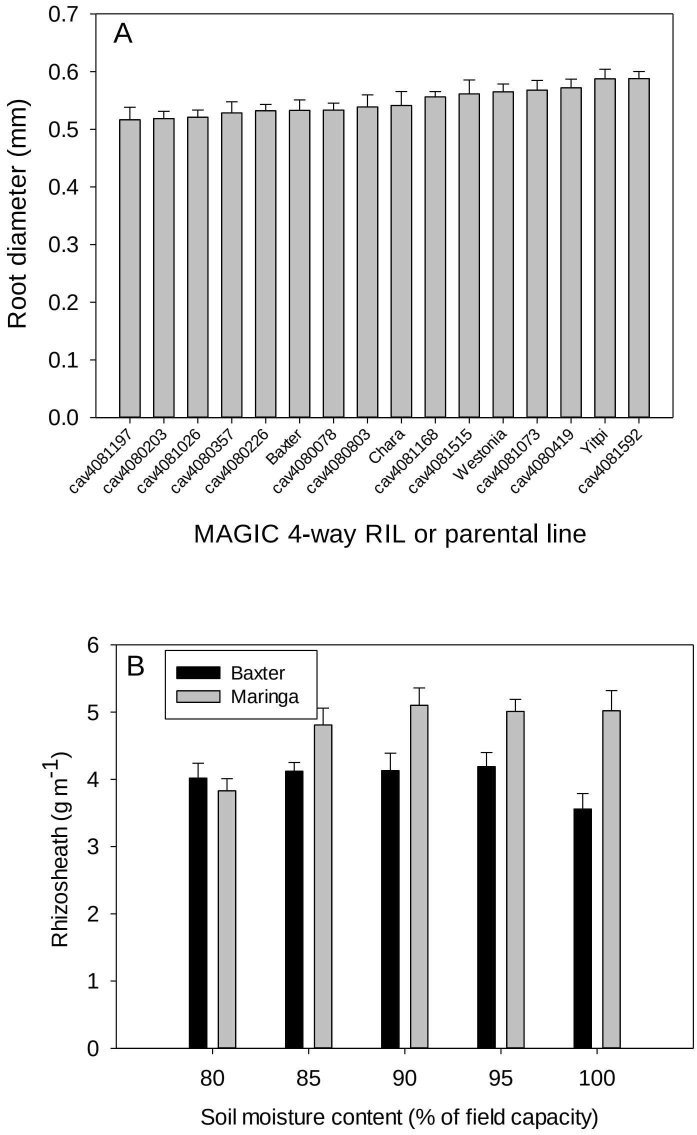

Supplementary Figure S1.

Root diameters of the parental lines and a set of random RILs taken from the 4-way MAGIC population (A) and influence of soil moisture content on rhizosheath size (B). For (A) rhizosheaths were collected (roots and soil) after 3 days growth and roots washed, photographed and the primary seminal roots analysed by Image J to determine their diameters. For (B) seedlings of two wheat cultivars with contrasting rhizosheaths (cv Baxter: small; cv Maringa: large) were grown in pots at the various soil moistures established at the start of growth and ~10 ml water added to the surface of the soil after 2 days growth. Rhizosheaths were measured after 3 days growth as described in the Methods.

Table S1

All QTL identified as contributing to formation of rhizosheaths including those with LOD < 3.0

Chromosome	Closest Marker	dist (cM)	% contibution to variation	LOD
1B	GENE-3626_308	51.82	2.6	2.47
2B	Tdurum_contig14482_423	101.39	5.3	4.8
3A	BobWhite_c12428_371	67.4	1.9	2.3
3A	BobWhite_c13210_115	205.45	1.4	1.36
3A	Kukri_c54593_543	292.59	2.4	2.69
4A	wsnp_Ex_c28728_37832012	198.1	3.1	2.68
4B	BS00010830_51	65.38	1.2	1.19
4D	wsnp_Ex_rep_c67296_65839761	31.79	4.5	4.79
5A	IACX5879	127.32	5.6	4.9
5B	wsnp_Ku_c12562_20256747	101.11	1.2	1
5B	BS00068710_51	138.61	7.2	5.92
5B	Tdurum_contig82077_129	166.82	2	1.75
6A	BobWhite_c15802_720	16.51	2.7	2.28
6A	wsnp_Ex_c11621_18716254	36.04	1.8	1.37
6A	IAAV7384	75.41	9.4	9.65
6B	wsnp_Ra_c3766_6947263	73.37	2.1	2.09
7A	BobWhite_rep_c49790_351	124.15	9.7	9.62
7B	wsnp_BF291608B_Ta_2_1	70.74	1.9	2.15
		Sum %var	66	

Table S2. Sequences producing significant alignments to *OsRHL1* with a BLASTN search of the IGWSC chromosomal arm survey sequences. Highlighted contigs indicate those that locate to the same chromosomal arms as rhizosheath QTL.

	Score(Bits)	E Value
IWGSC_chr6DL_ab_k71_contigs_longerthan_200_416241 ...	187	6e-44
IWGSC_chr6BL_ab_k71_contigs_longerthan_200_4258782 ...	181	3e-42
IWGSC_chr6AL_ab_k71_contigs_longerthan_200_5747359 ...	181	3e-42
IWGSC_chr5BL_ab_k71_contigs_longerthan_200_10874439...	163	7e-37
IWGSC_chr7AS_ab_k71_contigs_longerthan_200_4102091 ...	127	5e-26
IWGSC_chr5DL_ab_k71_contigs_longerthan_200_4494114 ...	125	2e-25
IWGSC_chr5AL_ab_k95_contigs_longerthan_200_1798755 ...	125	2e-25
IWGSC_chr6DL_ab_k71_contigs_longerthan_200_623134 ...	105	2e-19
IWGSC_chr7AS_ab_k71_contigs_longerthan_200_4127772 ...	93.3	1e-15
IWGSC_chr6BL_ab_k71_contigs_longerthan_200_4292512 ...	86.0	1e-13
IWGSC_chr7DS_ab_k71_contigs_longerthan_200_3924466 ...	84.2	5e-13
IWGSC_chr7BS_ab_k71_contigs_longerthan_200_3153924 ...	84.2	5e-13
IWGSC_chr6DL_ab_k71_contigs_longerthan_200_3261688 ...	84.2	5e-13
IWGSC_chr6DL_ab_k71_contigs_longerthan_200_3310669 ...	80.6	6e-12
IWGSC_chr6AL_ab_k71_contigs_longerthan_200_5826310 ...	80.6	6e-12
IWGSC_chr6BL_ab_k71_contigs_longerthan_200_4347612 ...	75.2	3e-10
IWGSC_chr6AL_ab_k71_contigs_longerthan_200_5789489 ...	75.2	3e-10
IWGSC_chr1DS_ab_k71_contigs_longerthan_200_1896224 ...	75.2	3e-10
IWGSC_chr1BS_ab_k71_contigs_longerthan_200_3473017 ...	75.2	3e-10
IWGSC_chr6DL_ab_k71_contigs_longerthan_200_3265172 ...	73.4	9e-10
IWGSC_chr6DL_ab_k71_contigs_longerthan_200_3230715 ...	73.4	9e-10
IWGSC_chr5DS_ab_k71_contigs_longerthan_200_2762657 ...	71.6	3e-09
IWGSC_chr5BS_ab_k71_contigs_longerthan_200_2244954 ...	71.6	3e-09
IWGSC_chr6AL_ab_k71_contigs_longerthan_200_5759916 ...	69.8	1e-08
IWGSC_chr5BS_ab_k71_contigs_longerthan_200_2236411 ...	69.8	1e-08
IWGSC_chr4BS_ab_k71_contigs_longerthan_200_4914721 ...	69.8	1e-08
IWGSC_chr2AL_ab_k71_contigs_longerthan_200_6368048 ...	69.8	1e-08
IWGSC_chr6DL_ab_k71_contigs_longerthan_200_3325852 ...	68.0	4e-08
IWGSC_chr6AS_ab_k71_contigs_longerthan_200_4359078 ...	68.0	4e-08
IWGSC_chr6AL_ab_k71_contigs_longerthan_200_5832796 ...	68.0	4e-08
IWGSC_chr5BL_ab_k71_contigs_longerthan_200_10811466...	68.0	4e-08
IWGSC_chr6DL_ab_k71_contigs_longerthan_200_3309722 ...	66.2	1e-07
IWGSC_chr5AS_ab_k95_contigs_longerthan_200_1517992 ...	66.2	1e-07
IWGSC_chr3B_ab_k71_contigs_longerthan_200_10500873 ...	66.2	1e-07
IWGSC_chr2AS_ab_k71_contigs_longerthan_200_5204747 ...	66.2	1e-07
IWGSC_chr7DS_ab_k71_contigs_longerthan_200_3963698 ...	64.4	5e-07
IWGSC_chr7AS_ab_k71_contigs_longerthan_200_4245279 ...	64.4	5e-07
IWGSC_chr3B_ab_k71_contigs_longerthan_200_10707949 ...	64.4	5e-07
IWGSC_chr2DL_ab_k71_contigs_longerthan_200_9898327 ...	64.4	5e-07
IWGSC_chr2DL_ab_k71_contigs_longerthan_200_9877687 ...	64.4	5e-07
IWGSC_chr2DL_ab_k71_contigs_longerthan_200_9853032 ...	64.4	5e-07
IWGSC_chr2DL_ab_k71_contigs_longerthan_200_9851928 ...	64.4	5e-07
IWGSC_chr6BL_ab_k71_contigs_longerthan_200_4254853 ...	62.6	2e-06
IWGSC_chr7DL_ab_k71_contigs_longerthan_200_3312422 ...	60.8	6e-06
IWGSC_chr5DL_ab_k71_contigs_longerthan_200_4549919 ...	60.8	6e-06
IWGSC_chr5BL_ab_k71_contigs_longerthan_200_10820313...	60.8	6e-06
IWGSC_chr3B_ab_k71_contigs_longerthan_200_10751654 ...	60.8	6e-06
IWGSC_chr3AL_ab_k71_contigs_longerthan_200_54442 3...	60.8	6e-06
IWGSC_chr1DL_ab_k95_contigs_longerthan_200_2246694 ...	60.8	6e-06

BLASTN 2.2.28+

Reference:
Stephen F. Altschul, Thomas L. Madden, Alejandro A. Schäffer, Jinghui Zhang, Zheng Zhang, Webb Miller, and David J. Lipman (1997), "Gapped BLAST and PSI-BLAST: a new generation of protein database search programs", Nucleic Acids Res. 25:3389-3402.
